# Negative-Pressure Pulmonary Edema and Takotsubo Cardiomyopathy in the Older Adults

**DOI:** 10.7759/cureus.22661

**Published:** 2022-02-27

**Authors:** Takahiro Sakamoto, Rie Sato, Akihiro Endo, Yoshiaki Iwashita, Kazuaki Tanabe

**Affiliations:** 1 Cardiology, Shimane University Faculty of Medicine, Izumo, JPN; 2 Emergency and Critical Care Medicine, Shimane University Faculty of Medicine, Izumo, JPN

**Keywords:** upper airway obstruction, heart failure, elderly women, takotsubo cardiomyopathy (ttc), negative pressure pulmonary edema

## Abstract

Negative-pressure pulmonary edema (NPPE) is a non-cardiogenic pulmonary edema caused by a sudden drop in the intrathoracic pressure associated with upper airway obstruction. Takotsubo cardiomyopathy (TCM) can often be triggered by intense stress and is more common in older women. In this case report, we describe a case of NPPE associated with upper airway obstruction and TCM associated with stress by upper airway obstruction in an 85-year-old woman. When encountering pulmonary edema associated with upper airway obstruction in older adults, the possibility of NPPE and TCM complications should be considered.

## Introduction

Negative-pressure pulmonary edema (NPPE) is a potentially life-threatening complication that occurs rapidly after upper airway obstruction. If the airway and respiratory management are appropriate during the acute phase, patients often recover without specific treatment; however, delays in the diagnosis and treatment can be fatal. In addition, cardiac complications should be considered. Takotsubo cardiomyopathy (TCM) is a condition caused by sudden stress. There are few reports of NPPE associated with TCM. In this case report, we describe the complications of NPPE and TCM in an 85-year-old woman with upper airway obstruction.

## Case presentation

**Figure 1:**
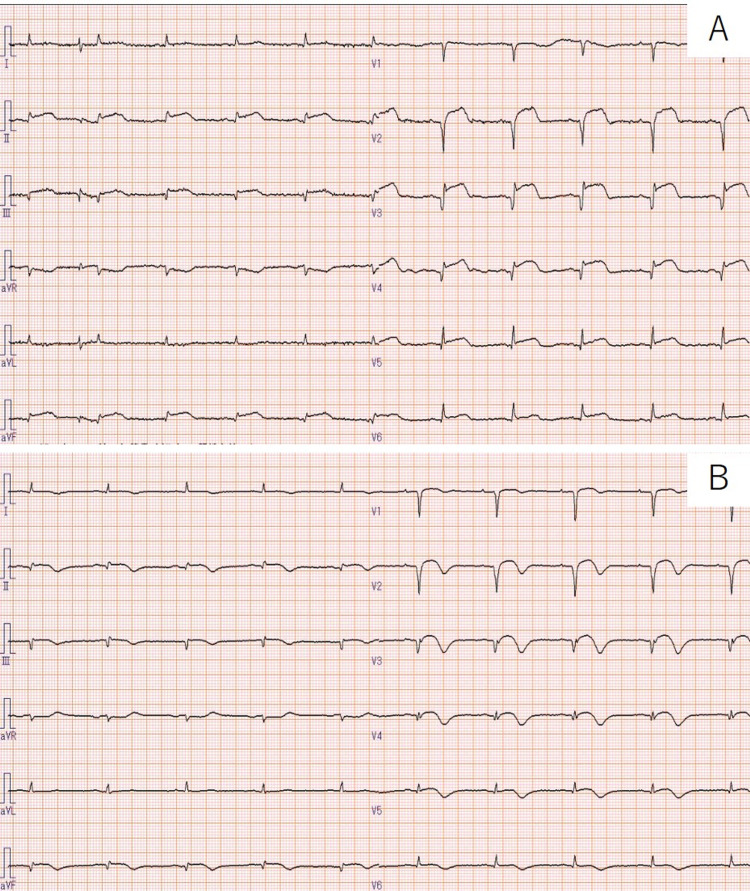
(A) The electrocardiogram (ECG) shows ST-segment elevation. (B) On her 11th day of hospitalization, the ECG shows negative T waves.

**Figure 2:**
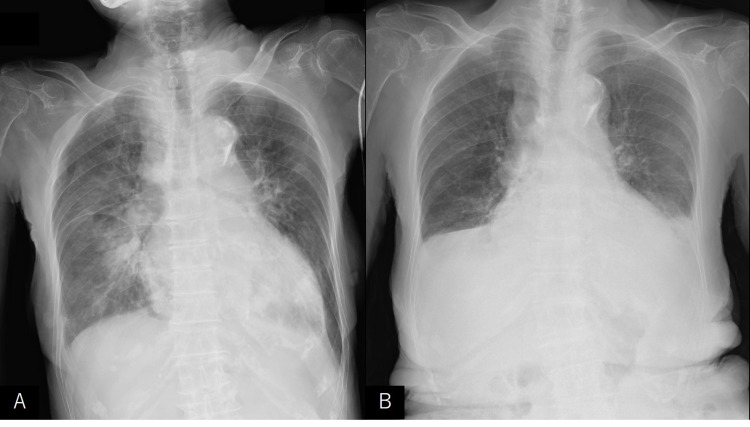
(A) Initial x-ray shows diffuse alveolar infiltrates throughout both lungs. (B) The pulmonary edema also improved on the sixth day of hospitalization. On the contrary, pleural effusion remained.

An 85-year-old woman with a history of dementia was choking in front of her family while eating, and her family members called the emergency helpline. When the emergency team arrived, her oxygen saturation was approximately 60% on 10 L O_2_; however, after removing a 5-6 cm shiitake mushroom from her mouth, her oxygen saturation improved, and she was able to respond. She was transported to our hospital by an ambulance, and her vitals at the time of arrival were as follows: body temperature, 36.1°C; blood pressure, 123/69 mmHg; heart rate, 65 beats/min; oxygen saturation, 98% on 10 L O_2_; and respiratory rate, 24 breaths/min. The electrocardiogram (ECG) demonstrated ST-segment elevation (Figure 1, Panel A). Blood tests showed an elevated troponin I of 1.52 ng/mL (the normal reference range < 0.04 ng/mL) but no increase in creatine kinase. Brain natriuretic peptide was elevated at 1,300 pg/mL. Chest x-ray revealed prominent pulmonary edema (Figure 2, Panel A). Echocardiography showed akinesis at the apex and hypercontractility at the base, suggesting TCM. Left ventricular ejection fraction was more than 50%, and there was no valvular disease. The early to late diastolic transmitral flow velocity (E/A) demonstrated an abnormal relaxation pattern, and no elevation tricuspid regurgitation peak gradient was observed. Urgent catheterization was considered necessary to differentiate from myocardial infarction. However, it was judged to be not a suitable candidate due to advanced dementia; therefore, diuretic therapy was started. Her family did not wish to have any invasive tests or treatments for her.

Within 24 hours, our patient no longer required oxygen. The pulmonary edema also improved on the sixth day of hospitalization (Figure 2, Panel B). On the contrary, pleural effusion remained, and diuretics were continued. On her 11th day of hospitalization, the ECG showed negative T waves (Figure 1, Panel B). Echocardiography demonstrated a slight improvement in asynergy at the apex, and there was no increase in troponin I levels. After rehabilitation, the patient was discharged without any chest symptoms after 24 days of hospitalization.

## Discussion

We encountered a complication of NPPE and TCM in an older woman. NPPE is a condition resulting in acute pulmonary edema after upper airway obstruction and was first described in 1973 [1]. The mechanism of NPPE is believed to be as follows: Markedly negative intrathoracic pressure is generated by deep inspiratory efforts against an occluded airway or a closed glottis. This negative intrathoracic pressure results in the augmentation of venous return to the right side of the heart and increased pulmonary venous pressure while decreasing the perivascular interstitial hydrostatic pressure. This favors the movement of fluid from the pulmonary capillaries into the interstitium and alveolar spaces, leading to the development of edema [2].

The most commonly reported etiology of NPPE in adults is laryngospasm during the intubation or postoperative period after anesthesia [2]. The literature suggests that it occurs more commonly than what is generally believed, with a frequency of 0.05%-0.1% of all anesthetic procedures, and is often unrecognized or misdiagnosed [3]. Treatment of NPPE depends on its severity and cause. In mild cases, oxygen therapy alone may be sufficient. NPPE is considered to recover within 24 hours with proper diagnosis and treatment; however, its mortality rate ranges from 11% to 40% if the diagnosis and treatment are delayed [4].

NPPE by choking does not occur without markedly negative intrathoracic pressure and is, therefore, more common in younger patients. However, there is a report of NPPE after upper airway obstruction caused by a sandwich in an older patient [5]. In our case, it was caused by shiitake mushrooms, a food item that is similar to a lid blocking the airway, and it is believed that this food item suddenly came in contact with the airway and blocked it completely, resulting in the development of NPPE. Furthermore, despite the sudden development of pulmonary edema, the patient improved after administering oxygen within 24 hours, which was considered consistent with the course of NPPE.

TCM is typically characterized by transient systolic dysfunction of the apical and mid-segments of the left ventricle in the absence of obstructive coronary artery lesions [6]. TCM is caused by physical or emotional stress. Recently, stress-induced TCM related to coronavirus disease 2019 has also been reported [7,8]. In our case, choking was the trigger of TCM. There have been reports of NPPE cases combined with TCM with an age range of 30-60 years [9-11]. To our knowledge, ours is a rare report of TCM in older adults. Although the precise mechanisms by which each of these pathologies occurs have not been delineated, Harmon et al. suspected that upper airway obstruction alone or in combination with NPPE generates a profound catecholamine surge, which may be essential in triggering TCM [9]. If NPPE alone is present, early resolution of symptoms is expected. However, if TCM is also present, treatment for heart failure should be considered. In our case, oxygen administration was no longer necessary at an early stage, but the prolonged diuretic course was required due to the residual pleural effusion caused by an additional cardiogenic pulmonary edema exacerbating the NPPE. Pleural effusion was also more common in patients with right ventricular involvement in TCM [12]. In this case, echocardiography did not clearly show right ventricular dysfunction, but it might have been revealed if MRI had been performed.

Although coronary artery evaluation is usually required to diagnose TCM, it could not be performed in our case due to dementia. Myocardial scintigraphy and coronary computed tomography angiography were also considered for the diagnosis of TCM, but they were not performed because the patient had difficulty giving consent due to dementia and the family did not wish to have them performed. However, based on the patient's history of sudden stress, lack of cardiovascular risk, akinesis of the apical segment as observed using echocardiography, elevated troponin T levels, and ECG changes, it was comprehensively determined that the patient had TCM.

## Conclusions

NPPE is a life-threatening complication that occurs rapidly after upper airway obstruction such as choking. NPPE is often unrecognized or misdiagnosed. NPPE due to choking is more common in younger patients. However, it may also occur in older adults, which may be complicated by TCM induced by intense stress. When NPPE due to choking is suspected in an elderly adult, not only the treatment of NPPE but also the differentiation of TCM and treatment for heart failure should be considered. The changes of ECG and echocardiography may be helpful for diagnosis.
